# Spheroids derived from the stromal vascular fraction of adipose tissue self-organize in complex adipose organoids and secrete leptin

**DOI:** 10.1186/s13287-023-03262-2

**Published:** 2023-04-07

**Authors:** Fermín Robledo, Lila González-Hodar, Pablo Tapia, Ana-María Figueroa, Fernando Ezquer, Víctor Cortés

**Affiliations:** 1 Department of Nutrition, Diabetes, and Metabolism, School of Medicine, Pontificia Universidad Católica de Chile, Santiago, Chile; 2 Center for Regenerative Medicine, School of Medicine, Clínica Alemana Universidad del Desarrollo, Santiago, Chile

**Keywords:** Organoid, Spheroid, Adipose tissue, Leptin, Adipogenesis

## Abstract

**Background:**

Adipose tissue-derived stromal vascular fraction (SVF) harbors multipotent cells with potential therapeutic relevance. We developed a method to form adipose spheroids (AS) from the SVF with complex organoid structure and enhanced leptin secretion upon insulin stimulation.

**Methods:**

SVF was generated from the interscapular brown adipose tissue of newborn mice. Immunophenotype and stemness of cultured SVF were determined by flow cytometry and in vitro differentiation, respectively. Spheroids were generated in hanging drops and non-adherent plates and compared by morphometric methods. The adipogenic potential was compared between preadipocyte monolayers and spheroids. Extracellular leptin was quantified by immunoassay. Lipolysis was stimulated with isoprenaline and quantified by colorimetric methods. AS viability and ultrastructure were determined by confocal and transmission electron microscopy analyses.

**Results:**

Cultured SVF contained Sca1 + CD29 + CD44 + CD11b- CD45- CD90- cells with adipogenic and chondrogenic but no osteogenic potential. Culture on non-adherent plates yielded the highest quantity and biggest size of spheroids. Differentiation of AS for 15 days in a culture medium supplemented with insulin and rosiglitazone resulted in greater *Pparg*, *Plin1,* and *Lep* expression compared to differentiated adipocytes monolayers. AS were viable and maintained leptin secretion even in the absence of adipogenic stimulation. Glycerol release after isoprenaline stimulation was higher in AS compared to adipocytes in monolayers. AS were composed of outer layers of unilocular mature adipocytes and an inner structure composed of preadipocytes, immature adipocytes and an abundant loose extracellular matrix.

**Conclusion:**

Newborn mice adipose SVF can be efficiently differentiated into leptin-secreting AS. Prolonged stimulation with insulin and rosiglitazone allows the formation of structurally complex adipose organoids able to respond to adrenergic lipolytic stimulation.

**Supplementary Information:**

The online version contains supplementary material available at 10.1186/s13287-023-03262-2.

## Introduction

Adipose stromal vascular fraction (SVF) is a heterogeneous and not fully characterized cell population [1, 2] that includes plastic-adherent mesenchymal stem cells reminiscent of those obtained from the bone marrow and umbilical cord cell suspensions [3–5]. Adipose tissue-derived stromal cells (ASCs) have self-renewal capacity and can be differentiated into various mesenchymal lineages. ASCs have been studied for tissue engineering and cell therapy due to their anti-inflammatory, antifibrotic, antiapoptotic, and proangiogenic activities [3, 6–8].

Classic mesenchymal stem cells (MSC) can self-organize as three-dimensional (3D) aggregates called multicellular spheroids. These structures have reportedly greater multilineage potential, secretion of therapeutic factors, and resistance against hypoxia compared to MSC cultured in monolayers (2D) [9–11]. Also, 3D cultures have cell-to-cell and cell-to-extracellular matrix interactions that allow them to reach cellular density, morphology, and functionalities that are more similar to animal tissues [12–14]. Although various 3D culture methods are available, the production of spheroids with scales, reproducibility, and costs suitable for clinical use, is still suboptimal [15]. Nevertheless, the therapeutic effects of MSC spheroids have been assessed in myocardial, vascular, bone, and glandular tissue regeneration, as well as in models of limb ischemia and wound healing [16].

Previous studies have demonstrated the feasibility of developing AS [17, 18]; however, only a few of them have shown adipokines gene expression or secretion [17, 19–21]. No study has assessed AS internal ultrastructure nor their functional response to lipolytic stimuli yet.

Herein, we report a method to form AS from the SVF of the interscapular adipose tissue of newborn mice, which maximizes leptin secretion and lipolytic response in comparison with traditional monolayers of differentiated adipocytes. We found that the AS generated by this method have a complex structure, with a multilayer organoid architecture and prolonged cell viability.

## Material and methods

### Raw data and details on protocols and materials are fully available upon request to the corresponding author (Víctor Cortés, vcortesm@uc.cl)

#### Animals

*Agpat2*^+/+^ neonatal mice with mixed C57BL6/J and 126 backgrounds [22] were used for SVF isolation, spheroid formation, and adipogenic differentiation. *Agpat2*^+/+^ mice are morphologically normal and develop no metabolic abnormalities. By contrast, *Agpat2*^−/−^ littermates develop lipodystrophy and were used for alternative investigations in the laboratory. Mice were kept with 12-h light and dark cycle, free access to water, and a standard chow diet. In total, 64 newborn mice, corresponding to 7 independent litters, were used in the present study. Animal procedures were approved by the animal safety and bioethics committee of Pontificia Universidad Católica de Chile ((1) Title of the approved project: “Terapia celular para la lipodistrofia congénita generalizada tipo 1”; (2) Name of the institutional approval committee or unit: Comité Ético Científico para el Cuidado de Animales y Ambiente; (3) Approval number: 190531003; (4) Date of approval June 19, 2021).

#### Isolation and culture of the stromal vascular fraction (SVF) from the interscapular adipose tissue of newborn mice

Newborn mice were euthanized by decapitation, and interscapular adipose tissue was surgically resected, rinsed with phosphate-buffered saline (PBS), and incubated with digestion buffer (300 µl, 0.2% collagenase type II in 25 mM KHCO3 buffer, 12 mM KH2PO4, 1.2 mM MgSO4, 4.8 mM KCl, 120 mM NaCl, 1.2 mM CaCl2, 5 mM glucose, 2.5% BSA and 1% penicillin/streptomycin, pH 7.4) for 45 min at 37 °C. The digestion product was passed through a 100-µm mesh. ACK buffer (500 µl, 4 min at room temperature) was added to eliminate blood cells and centrifuged at 300 g for 5 min. Pelleted cells were resuspended in culture medium (1 ml, DMEM F12 10% FBS, 1% Antibiotic–Antimycotic (Gibco, #cat 15,240,062), pH 7.2) and filtered through a 40-µm mesh, followed by centrifugation, resuspension in culture medium and seeded in 24-well plates. Upon confluence, cells were detached with 0.25% trypsin for 5 min and seeded for expansion in a culture surface three times the original area, repeating this process for 3 rounds in total. Cells were kept in an incubator at 37 °C with 5% CO_2_.

#### Immunophenotyping of cultured SVF

For immunophenotyping, after two passages 50,000 cells/ml were incubated with unconjugated anti-CD16/CD32 antibody (eBioscience, #cat. 14,016,185) diluted in αMEM supplemented with 5% FBS medium for 20 min on ice, followed by 45-min incubation with anti-Sca1-APC-conjugated (eBioscience, #cat. 175,981), CD29-FITC-conjugated (eBioscience, #cat. 110,291), CD44-PE-Cy5-conjugated (BD Pharmingen, #cat. 553,135), CD90-PE-conjugated (eBioscience, #cat. 12,090,081), CD11b-efluor660-conjugated (eBioscience, #cat. 500,110) or CD45 (BD Pharmingen) antibodies, for flow cytometry analysis (CYAN ADP, Dako Cytomation, Carpinteria, CA). Summit V 4.3 software was used for data acquisition and processing. Cells labeled with the corresponding isotype antibodies were used as autofluorescence controls.

#### Osteogenic and chondrogenic differentiation

Differentiation potential was determined as previously described [23]. For osteogenic differentiation, SVF was seeded in plastic dishes with a density of 25,000 cells/cm^2^ in αMEM medium supplemented with 10% FBS and 80 µg/ml gentamicin and cultured for 24 h. Osteogenic differentiation was induced with αMEM supplemented with 10% FBS, 80 µg/ml gentamicin, 0.1 µM dexamethasone, 50 µg/ml 2-phosphate ascorbate, and 10 mM β-glycerol phosphate. This medium was changed twice a week, and the formation of hydroxyapatite deposits was assessed with alizarin red staining after 18 days of osteogenic induction. For chondrogenic differentiation, 50,000 cells were concentrated in 10 µl of αMEM supplemented with 10% FBS and 80 µg/ml gentamicin. Cell agglomerates were incubated in the center of a 1.9 cm^2^ culture well for 2 h. Chondrogenic differentiation was induced with αMEM supplemented with 10% FBS, 80 µg/ml gentamicin, 0.1 µM dexamethasone, 50 µg/ml ascorbate 2-phosphate, 0.5 U/ml insulin and 10 ng/ml TGF-β3. Differentiation media was changed twice a week, and the formation of glycosaminoglycans was verified with 1.7 safranin-O staining at the day 10 of differentiation.

#### Alizarin red staining

Cultures subjected to osteogenic differentiation were fixed with 70% ethanol, rinsed with PBS, and incubated with 40 mM alizarin red for 10 min at room temperature. After rinsing with double distilled water, cells were incubated with PBS for 15 min at room temperature. Stained cultures were visualized and photographed using a bright field microscope.

#### Safranin-O staining

Cells were fixed in 70% ethanol and incubated in 0.1% safranin-O for 5 min at room temperature and serially rinsed with double distilled water, 70% ethanol, and 100% ethanol. Stained cultures were visualized and photographed using a bright field microscope.

#### Spheroid formation in hanging drops

Previously expanded SVF cultures were serially diluted from a starting concentration of 750,000 cells/ml (DMEM-F12 supplemented with 10% FBS and 1% antibiotic-antifungal). Ten microliters of drops containing 7,500, 3,750, or 1,875 cells, respectively, was deposited on the internal surface of an inverted cap (96-well plate). Additionally, a single 50-µl PBS drop was placed in the bottom of each well for humidification purposes. Caps loaded with drops were carefully replaced on the plates and incubated for 48 h to allow cells aggregation by gravity. Only 60 drops were created per 96-well plate because peripheral wells were kept unused due to the higher evaporation rates compared to central wells. Cells were maintained at 37 °C, with a 5% CO_2_ atmosphere.

#### Spheroid formation on low-attachment plates

SVF cultures were serially diluted from a starting suspension of 750,000 cells/ml (DMEM-F12 supplemented with 10% FBS and 1% antibiotic-antifungal) to 375,000, 187,500, or 100,000 cells/cm^2^. Cells were seeded on low-attachment 24-well plates (SPL, #cat. 32,024) in 1 ml of DMEM-F12 supplemented with 10% FBS and 1% gentamycin/streptomycin. After 24 h, cultures were mechanically disaggregated by gentle pipetting, diluted (1:5) in culture medium, seeded in new low-attachment 24-well plates (SPL, #cat. 32,024) (500 µl per well), and incubated for additional 24 h. Spheroids were counted and transferred to 35-mm low-adhesion dishes (SPL, #cat. 11,035), with a maximum of 1,000 spheroids per dish. The medium was changed every 48 h, and floating spheroids were recovered by spontaneous decantation (2 min) in 15-ml conical tubes at room temperature. The medium was carefully removed by manual pipetting, and spheroids were resuspended in fresh culture medium and seeded on new low-attachment plates.

### Spheroids counting

To compare hanging drops and low-adherence plate method yields, spheroids were manually counted by bright-field microscopy after 48 h of culture. For this, spheroids from 4–10 hanging drops or 3–10 wells (24-well low-attachment plates) were pooled and decanted in 15-ml conical tubes, the medium was discarded, and the spheroids were resuspended in 1 to 5 ml of DMEM F12 SFB 10% medium. Three hundred microliters of spheroids suspension was transferred to a 35-mm mesh plate (SPL, #cat.110350) for counting and then recovered. The total number of spheroids was expressed relative to the resuspension volume. Spheroids’s concentration was adjusted to 1,000 spheroids/ml. From this suspension, 1 ml was used for mRNA extraction, 200 µl for cryosection, and 100 µl for leptin quantification or microscopy analyses.

### Spheroids size determination

Spheroid size (feret) was determined at 48 h of formation. Spheroids formed in 20–30 hanging drops, or 1–3 wells of 24-well low-attachment plates, were photographed using a bright-field microscope with 10 × magnification, from 2 independent experiments. Photographs were analyzed by batch analysis using a custom-designed macro in image J software (Additional file 1: Table S1). Image processing included scaling, luminance correction and threshold-based binary transformation in spheroids with feret equal to or greater than 50 µm.

### Comparison between cultured monolayers and spheroids

SVF cultures from individual newborn mice were adjusted to 750,000 cells/ml. Cultures were partitioned in a 24-well low attachment plate for spheroid formation (1 ml per well), a 24-well regular adherent plate for microscopy and leptin quantification of cultured monolayer (500 µl per well of a 1/10 dilution), and a 6-well regular adherent plate for RNA extraction of cultured monolayer (3 ml of a 2/15 dilution).

### Adipogenic differentiation of SVF monolayers and spheroids

SVF cultured in regular plastic plates (monolayers) and 48-h formed spheroids were differentiated into mature adipocytes with a two-phase protocol (herein named “i20”) previously used in our laboratory [24]. Adipogenesis was induced with DMEM-F12 supplemented with 10% FBS, 1% antibiotic-antifungal solution, 500 nM dexamethasone, 125 nM indomethacin, 0.5 mM IBMX, 1 nM rosiglitazone, 1 nM T3 and 20 nM insulin. After 48 h, induction medium was replaced by maintenance medium (DMEM-F12 supplemented with 10% FBS, 1% antibiotic–antimycotic solution, 1 nM T3 and 20 nM insulin). To increase leptin secretion, new protocols were tested. First, an insulin dose–response curve was performed with 0 nM (i0), 2,000 nM (i2,000) and 10,000 nM (i10,000) insulin, in both induction and maintenance phases. In the following experiments, 1 nM rosiglitazone was added into i2,000 maintenance medium. This latter procedure was dubbed “InRo”.

### Expression profile of mature adipocyte markers

Total RNA from a 6-well plate monolayer culture or a suspension of 1,000 spheroids was extracted with TRIzol™ (Invitrogen, #cat. 15,596,018), following manufacturer's instructions. Contaminant DNA was digested (TURBO DNAse, Invitrogen, #cat. AM1907), and total RNA was reverse-transcribed (Applied Biosystems, #cat. 4,368,814). The abundance of adipogenic markers Cebpb, Pparg, Plin1, Adipoq and Lep was quantified in 100 ng of cDNA (Step One thermocycler, Applied Biosystems). PCR primers are indicated in Additional file 2: Table S2. Relative expression was calculated with the ΔΔCt method with cyclophilin as reference gene.

### Leptin quantification

Leptin concentration in conditioned medium of differentiated monolayers (300 µl in 24-well plates) and AS (pool of 3 wells in 96-well plate with 100 spheroids each) was measured by ELISA (Mouse Leptin ELISA, Millipore, #cat. EZML-82 K). Cells were collected for DNA quantification (Easy-DNA™ gDNA Purification Kit, Invitrogen, #cat. K180001) to normalize leptin concentration by total DNA. Four biological replicates per group were analyzed.

### Lipolysis stimulation and glycerol quantification

50–100 AS were incubated for 3 h in color-free DMEM-F12 medium (Gibco, #cat. 21,041,025) with or without 1 μM isoprenaline (Sigma #cat. I5627). After incubation, the medium was collected for glycerol quantification with a colorimetric kit (Sigma, #cat TR0100). Spheroids were collected for gDNA extraction with Easy DNA™ kit to normalize glycerol measurement.

### Fluorescence microscopy

Floating living AS were incubated for 10 min in DMEM-F12 medium supplemented with 10% FBS and 1% antibiotic–antimycotic solution with Hoechst (1: 1,000), Bodipy (1: 1,000), and propidium iodide (1: 1,000) for determining nuclei, neutral lipids, and viable cells, respectively. AS were fixed with 4% buffered PFA for 24 h at room temperature. Fixed spheroids were decanted by centrifugation for 5 min at 300 g and rinsed 3 times with PBS. Images were captured by laser scanning confocal microscopy with an LSM 880 ZEISS microscope with Airyscan detection, with 1708 × 1708 resolution. Objective Plan-Apochromat 10 × NA 0,45; 32 channels GaAsP PMT spectral detector; filter set 38 GFP (EX 470/40 EM 525/50), filter set 43 Cy3 (EX 545/25 EM 605/70) and filter set 43 DAPI (EX 365 EM 445/50). Setting and configuration by ZEN 2.3 black and ZEN Blue software. Whole spheroids were analyzed by Z-stack imaging with heights in the range of 50 to 100 µm and a cross-sectional area of approximately 400 × 400 µm. Surface reconstruction of spheroids was made by Imaris Viewer software.

### Transmission electron microscopy

AS at day 15 of differentiation were fixed with 2.5% glutaraldehyde in 0.1 M sodium cacodylate buffer, pH 7.0, and incubated with 1% OsO4. AS were dehydrated with ethanol and infiltrated with Epon 812 epoxy resin. Ultrathin sections (80 nm) were obtained, and grids were visualized with Phillips Tecnai 12 (BioTwin) electron microscope and photographed with a CIS CCD Megaview G2 camera (Olympus Corp). Individual images corresponding to different fields were assembled to compose an overview of a single whole spheroid.

### Statistical analysis

Statistical analysis and plotting were performed with GraphPad Prism 8. The abundance of adipocyte markers and leptin secretion in monolayers and spheroids were compared by two-way ANOVA, considering a quantitative variable (the relative amount of specific mRNAs or leptin concentration) and two nominal variables (days of differentiation and culture format or differentiation protocol). Spheroids feret showed high heteroscedasticity (Brown-Forsythe; F(5,1427) = 88,2; *p* < 0,0001). To overcome this difficulty, log transformation was applied, and every combination of culture format and cell concentration was considered as an independent group, analyzed by Welch-ANOVA with Games–Howell multiple comparison post-test.

## Results

### Immunophenotype and differentiation potential of the stromal vascular fraction of newborn mice interscapular adipose tissue

The procedures for harvesting and culturing mesenchymal stem cells (MSC) [2, 25] are analogous to those reported for stromal vascular fraction/preadipocytes (SVF) [22, 24]; however, the phenotypical and functional overlapping between MSC, adipocyte stem cells (ASC) and preadipocytes remains controversial [26]. We assessed the cellular identity of newborn mice interscapular adipose tissue SVF following the minimal criteria for MSC identification defined by the International Society for Cell Therapy: (1) adhesion to plastic under standard culture conditions, (2) expression of putative MSC surface markers, and absence of surface markers characteristics of other lineages, and (3) ability to differentiate into osteoblasts, adipocytes, and chondroblasts [23]. Although a consensus for the immunophenotype of murine MSC is lacking, it is conventionally accepted that they must express Sca1, CD29, CD44, and CD90, while CD11b and CD45 must be absent [27, 28].

**Fig. 1:**
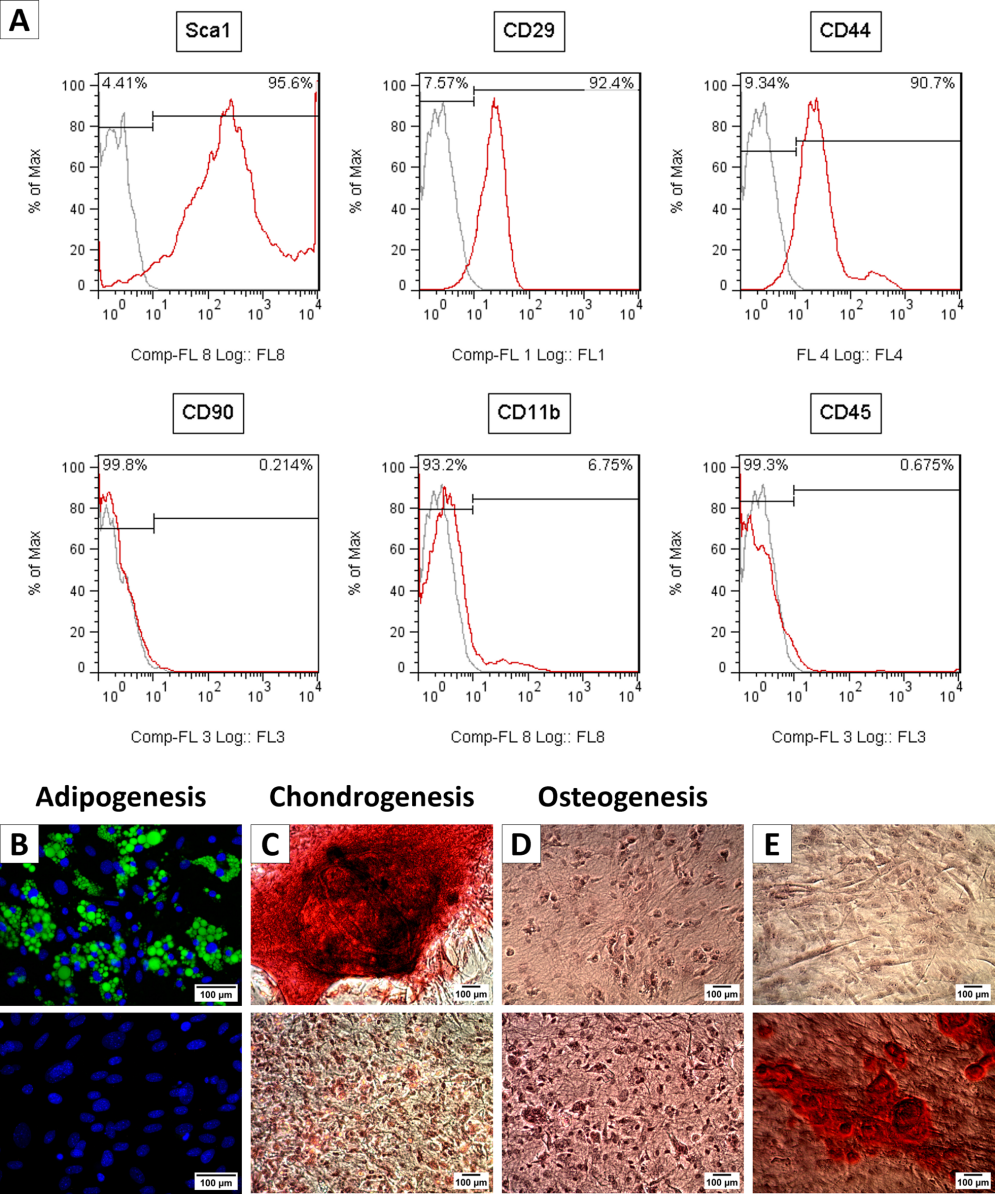
Characterization of primary culture derived from the stromal vascular fraction of interscapular adipose tissue of newborn mice. **A** Analysis by flow cytometry for expression of the Sca1, CD29, CD44, CD90, CD11b, and CD45 markers. In each panel, the red line represents the marker of interest, the gray line the isotype control, and the upper part indicates the percentage of the negative and positive populations for each marker. In vitro adipogenic (**B**), chondrogenic (**C**), and osteogenic (**D**) differentiation was assessed by oil Bodipy staining of neutral lipids, safranin-O staining of cartilage, and alizarin red staining of hydroxyapatite, respectively. In each case, the result of the differentiation is shown in the upper image and undifferentiated control in the lower image. **E** Corresponds to a positive control of osteogenic differentiation using human MSC stained with alizarin red

After two passages, SVF cultures were analyzed by flow cytometry. As shown in Fig. 1A, SVF from newborn mice interscapular adipose tissue was composed of cells expressing Sca1 (95.6%), CD29 (92.4%) and CD44 (90.7%), while expression of CD11b, CD45 and CD90 was either low (6.75%) or very low (0.68%; 0.21%), respectively. These results suggest that the cultured SVF used in this study is composed of mesenchymal cells with a low or very low contribution of hematopoietic cells. However, given the low expression levels of CD90, these cells do not strictly fit with MSC definition.

Next, we characterized the differentiation potential of newborn mice interscapular adipose tissue SVF. Previously, we have reported the differentiation potential of this SVF into both white and brown adipocyte-like cells [22, 24]. Herein, we confirmed adipocyte differentiation by lipid droplets buildup (Fig. 1B). Chondrogenic capacity [7, 29, 30], was also verified by the formation of cells stained with Safranin O (Fig. 1C). By contrast, our SVF was unable to undergo osteogenic differentiation as indicated by the absence of hydroxyapatite crystals laden cells (Fig. 1D), indicating that cultured SVF derived from the interscapular adipose tissue of newborn mice contains bipotential mesenchymal cells rather than properly multipotential MSCs. As a positive control of osteogenic differentiation we differentiated human MSC that efficiently accumulated hydroxyapatite crystals (Fig. 1E).

### Spheroids formation from the stromal vascular fraction of newborn mice interscapular adipose tissue

**Fig. 2:**
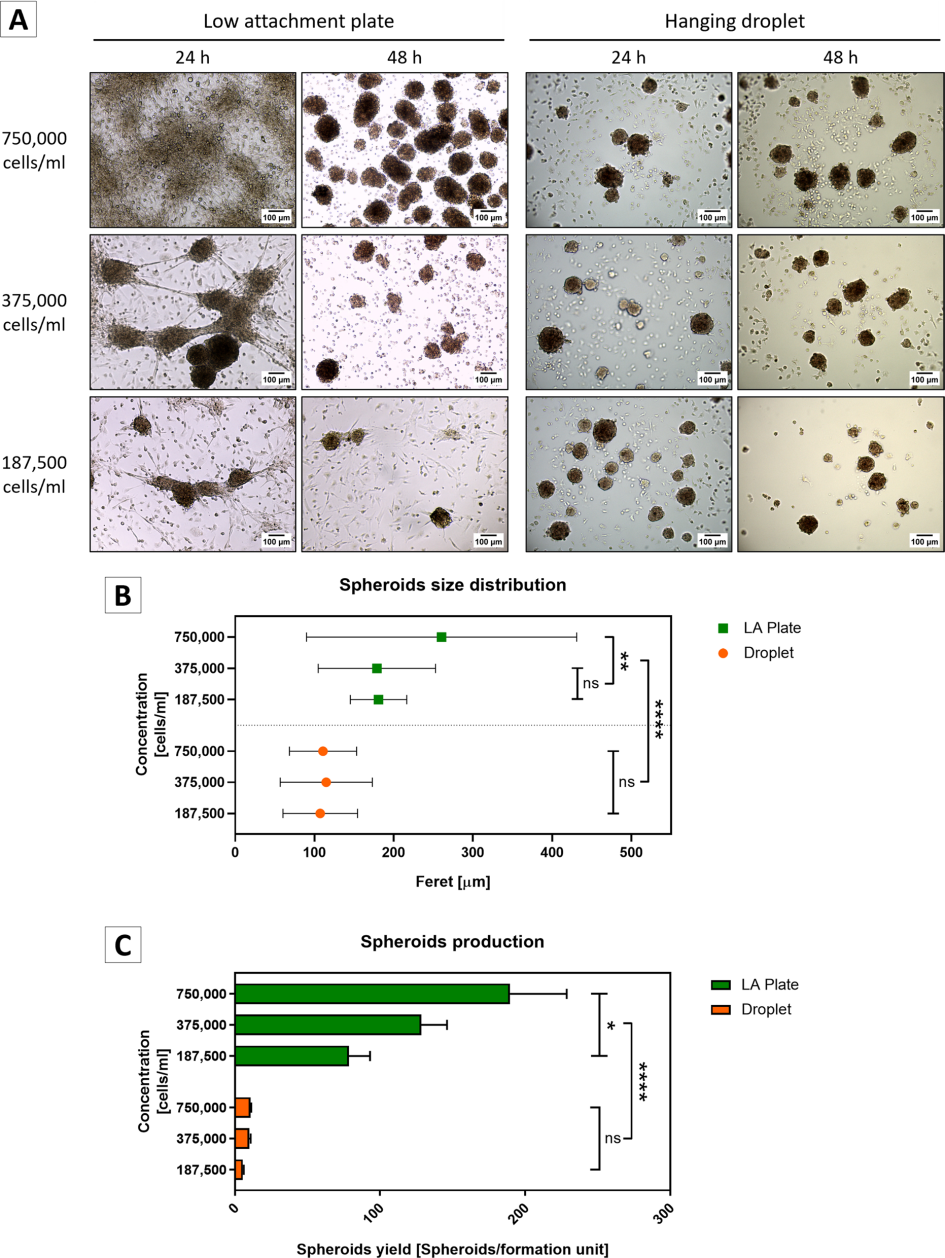
Formation of murine SVF-derived spheroids in low adherence plates and hanging drop cultures. **A** Serial dilutions of 750,000, 375,000, and 187,500 SVF cells/ml (from upper to bottom row) were seeded in low attachment plates (LA, left panels) or hanging drops (right panels), as indicated, and spheroids formation was checked after 24 and 48 h. Only in low attachment plate experiments, cultures were mechanically disaggregated after 24 h, diluted 1:5 and re-plated for additional 24-h incubation. All images were taken using a bright-field microscope with 10 × magnification. 100 µm scale bar size. **B** Spheroids formed in low attachment plates or hanging drop were photographed (bright field microscopy) and subjected to particle analysis with ImageJ software. The number of analyzed spheroids was: 1) hanging drop: 187,500 cells/ml *N* = 235; 375,000 cells/ml *N* = 234; 750,000 cells/drop *N* = 475; 2) low attachment plate: 187,500 cells/ml *N* = 37; 375,000 cells/ml *N* = 48; 750,000 cells/ml *N* = 404. Size distribution by calculation of feret expressed as mean and standard deviation of each population. Welch-ANOVA; W (5, 224) = 129.9; *p* < 0.0001; Games–Howell post-test. **C** Yield of spheroid formation per formation unit with 4 runs per group. Two-way ANOVA, Droplet vs LA Plate: F (1, 18) = 266.7; *P* < 0.0001; Initial cell concentration: F (2, 18) = 19.74; *P* < 0.0001; Tukey's post-test. * *p* < 0.05; ** *p* < 0.01; *** *p* < 0.001; **** *p* < 0.0001; ns not significant

First, we compared the efficiency of SVF spheroids formation using hanging drops and low-adherence methods. Serial dilutions (750,000, 375,000 and 187,500 cell/ml) of SVF were used as starting material. The hanging drops method produced homogeneous spheroids after 24 h in all cell dilutions tested (Fig. 2A). By contrast, the non-adherent plate method resulted in scarce isolated spheroids in cultures that were started with 187,500 cells/ml, whereas those started with 375,000 cells/ml produced a combination of isolated spheroids and heterogeneous cell aggregates. Cultures started with 750,000 cells/ml were able to generate a layer of irregular density with numerous cellular aggregates. Importantly, when these cultures were dispersed by pipetting, re-diluted, and seeded for an additional 24 h, they yield numerous isolated and well-formed spheroids (Fig. 2A).

Total yield and spheroids size were characterized at 48 h of formation with an automatized morphometric protocol (Additional file 1: Table S1). We found significant differences in spheroids feret (Welch-ANOVA test; W (5,288) = 138.8; *p* < 0.0001) between formation methods and SVF initial dilution. Spheroids formed in non-adherent plates ranged from 100 to 400 µm feret with those formed at an initial concentration of 750,000 cells/ml being the largest (260.4 ± 170.5 µm) (Fig. 2B). Importantly, it has been previously reported that spheroids up to 500 µm in diameter have proper oxygen and nutrient diffusion [31], suggesting that the spheroids formed with our procedures must have preserved viability and functionality.

Although the hanging drop method produced more homogeneous spheroids, the yield of each individual drop was low (~ 10 spheroids per drop). On the contrary, the non-adherent plate method allowed the formation of abundant and large spheroids. Therefore, we concluded that culturing 750,000 cells/ml in low adherence plates was the best method for generating SVF spheroids, with a mean of 189 spheroids per well in a 24-well plate (Fig. 2C). Considering that the interscapular adipose tissue of a single newborn mouse yield ~ 9.3 million SVF cells (data not shown), we estimated a total yield of ~ 2,433 spheroids per animal.

### Optimization of leptin secretion by differentiated adipose spheroids (AS)

Upon a classic adipogenic protocol (henceforth called “i20” because of 20 nM insulin concentration in the induction and maintenance media), monolayers of newborn mice interscapular adipose tissue SVF can be differentiated into adipocyte-like cells, reaching a maximum of 80% of lipid-laden cells between days 7 and 10 after adipogenic induction [24, 32].

Herein we found that adipogenic differentiation of SVF spheroids with i20 protocol also resulted in neutral lipid build-up and expression of markers of mature adipocytes (Additional file 3: Fig. S1A–B). However, adipokines gene expression, particularly leptin, was lower than in parallel cultures of differentiated SVF monolayers and in the white adipose tissue of adult mice (Additional file 3: Fig. S1B).

Insulin promotes leptin secretion in mature adipocytes [33–36]; however, the range of insulin concentrations used in the literature is extremely wide (0.2 to 12,052 nM) [37]. We tested the effect of different insulin concentrations (0 nM (i0 medium); 20 nM (i20 medium); 2,000 nM (i2,000 medium) or 10,000 nM (i10,000 medium)) on AS differentiation efficiency and leptin expression.

As expected, all markers increased in direct proportion to insulin concentration up to 2,000 nM insulin (Additional file 4: Fig. S2). Since PPARγ is the master regulator of adipogenesis [38], we tested the effect of adding the PPARγ agonist rosiglitazone during the early and late phases of differentiation. We reasoned that as PPARγ levels remain very low in AS differentiated with the i20 protocol, particularly during early differentiation phases (Additional file 3: Fig. S1A), stimulation with rosiglitazone only during the first 48 h after adipogenic induction, as used in classic adipogenic protocols, should be insufficient to promote maximal adipogenic differentiation. We also considered that rosiglitazone diffusion towards spheroid inner portions might require longer times to enable adipocyte differentiation in large spheroids. Therefore, we compared AS differentiated with i20 and i2,000 protocols with those formed with a modified i2,000 protocol, maintaining 1 nM rosiglitazone throughout the entire differentiation procedure (herein dubbed as "InRo" protocol). Adipogenic differentiation was monitored by quantifying mRNA levels of Cebpb, Pparg, Plin1, Adipoq and Lep.

**Fig. 3:**
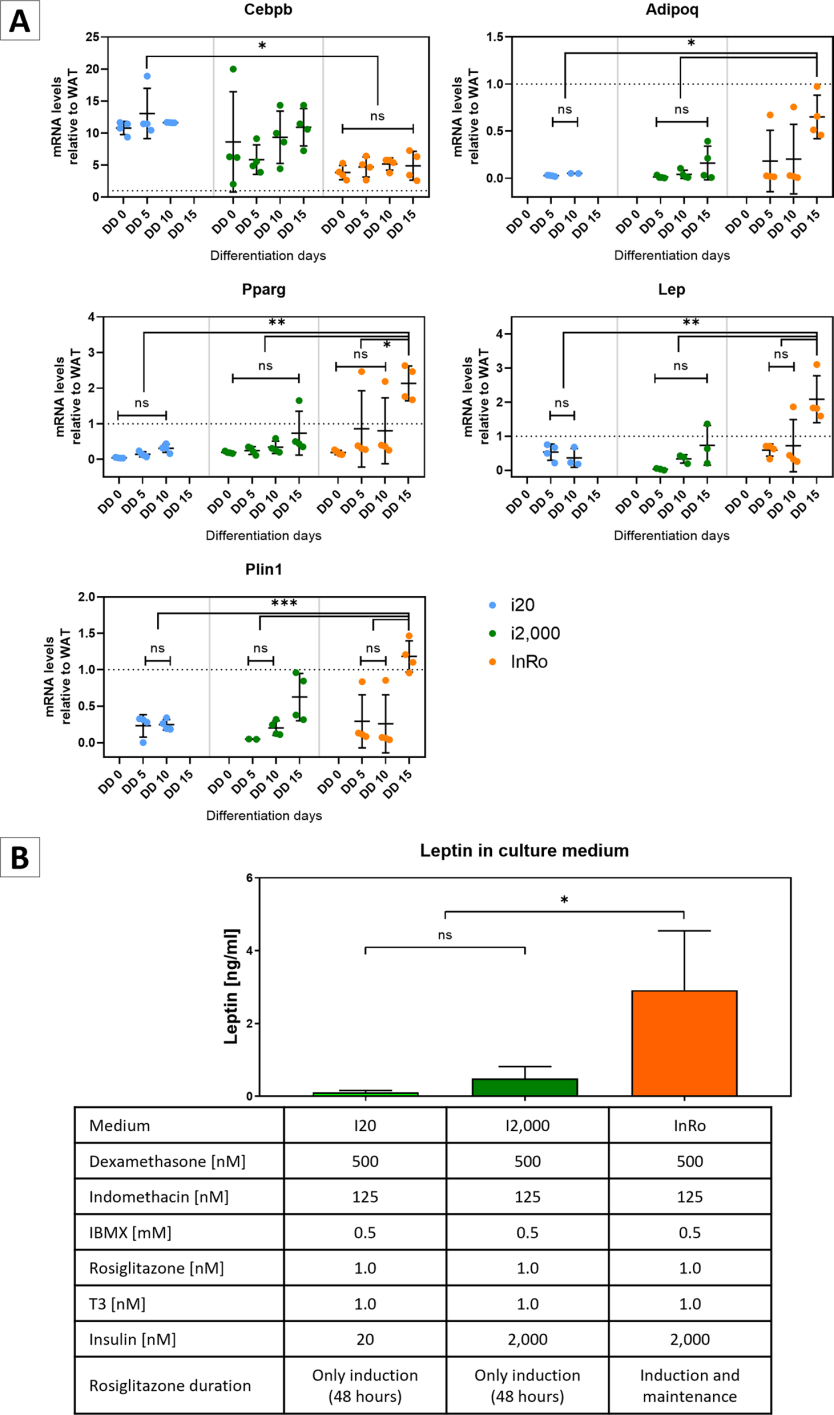
Effect of insulin and rosiglitazone on adipogenic differentiation and leptin secretion of AS. **A** Quantification of mRNA levels of Cebpb, Pparg, Plin1, Adipoq, and Lep relative to white adipose tissue (WAT) of adult mice (dotted line) in AS at 15 days of adipogenic differentiation with cocktail i20, i2.000, or InRo. Data are presented as mean ± standard deviation, *N* = 4 per group. Two-way ANOVA with Tukey's post-test. **B** Leptin concentration in conditioned medium (100 spheroids per group, 48 h) at day 15 of adipogenic differentiation. *N* = 3. One-way ANOVA (F (2, 6) = 7.539; *p* = 0.0231). * *p* < 0.05; ns: not significant

Adipocyte markers Pparg, Plin1, Adipoq and Lep were significantly increased along adipogenic differentiation with the three tested protocols (two-way ANOVA; Pparg, medium: F (2, 36) = 14,37 *P* < 0,0001; differentiation days (DD): F (3, 36) = 6,137 *P* = 0,0018; Interaction: F (6, 36) = 3,596 *P* = 0,0068; Plin1, medium: F (2, 25) = 9,513 *P* = 0,0008; DD: F (2, 25) = 10,03 *P* = 0,0006; interaction: F (4, 25) = 7,862 *P* = 0,0003; Adipoq, medium: F (2, 25) = 8,431 *P* = 0,0016; DD: F (2, 25) = 3,379 *P* = 0,0503; interaction: F (4, 25) = 2,040 *P* = 0,1194; Lep, medium: F (2, 23) = 12,95 *P* = 0,0002; DD: F (2, 23) = 5,061 *P* = 0,0151; F (4, 23) = 6,482 *P* = 0,0012) (Fig. 3A). However, the AS differentiated with InRo protocol showed the highest mRNA levels of Pparg (*P* < 0.05), Plin1 (*P* < 0.001), Adipoq (*P* < 0.01), and Lep (*P* < 0.01) at any time point of analysis, compared to those differentiated with i20 or i2,000 procedures (Fig. 3A). Importantly, leptin mRNA levels were even higher in AS differentiated with InRo protocol (day 15) compared with adult mice WAT (2.09 ± 0.69-fold change).

To assess adipokines secretion, we quantified leptin concentration in the conditioned medium of differentiated AS. Concordant with our gene expression analysis, we found that leptin concentration was significantly higher in the medium of InRo-differentiated AS compared to all other differentiation protocols (i20: 0,11 ± 0,06 ng/ml; i2,000: 0,49 ± 0,33 ng/ml; InRo: 2.92 ± 1.63 ng/ml) (one-way ANOVA, F (2, 6) = 7,539; *P* = 0,0231; post-Tukey test with *P* < 0.05 in all comparisons of InRo medium against i20 or i2.000) (Fig. 3B).

Thus, we found that the addition of rosiglitazone across adipogenic differentiation of AS (InRo protocol) determines increased abundance of mature adipocyte markers and leptin concentration in conditioned medium compared to protocols that restrict rosiglitazone to the induction phase only.

**Fig. 4:**
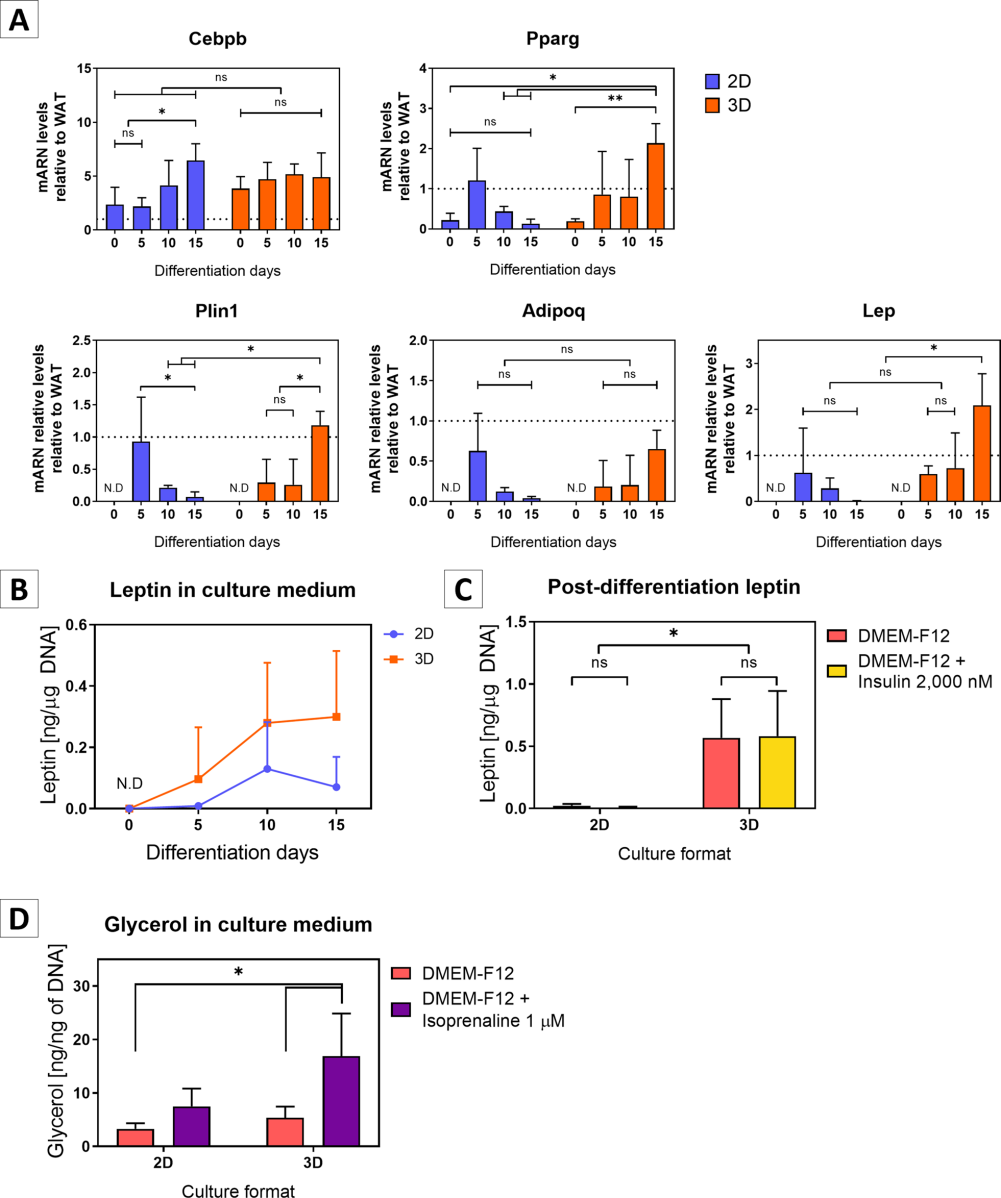
AS differentiated with InRo medium have increased expression of adipogenic markers and leptin secretion compared to adipocyte monolayers and increased glycerol secretion in response to isoprenaline stimulation. **A** Quantification of mRNA levels relative to adult mouse WAT (dotted line). Data are presented as mean ± standard deviation. *N* = 4 per group. Two-way ANOVA with Tukey's post-test. **B** Leptin concentration in conditioned medium (48 h) normalized to total DNA. Data are presented as mean ± standard deviation. *N* = 4 per group. **C** Differentiated adipocyte monolayers and AS at day 15 of differentiation were incubated with DMEM-F12 medium plus 10% SFB in the absence of insulin and rosiglitazone for 48 h. Next, cells were incubated for additional 48 h with DMEM-F12 medium plus 10% SFB in the presence or absence of 2,000 nM insulin. The conditioned medium (48 h) was harvested for leptin quantification and normalized by total DNA. Data are presented as mean ± standard deviation. *N* = 4 per group. A significant effect for culture format was determined (F (1, 12) = 21.93; *p* = 0.0005). **D** 2D adipocytes and AS were differentiated with InRo protocol and incubated by 3 h in color-free DMEM-F12 medium with or without 1 μM isoprenaline. Conditioned medium was collected for glycerol determination by colorimetric methods and AS. Cells were collected for total DNA extraction and quantification. Data are represented as mean ± standard deviation. *N* = 3 for monolayer cultures and 5 for AS groups. There was a significant increment in glycerol secretion in response to isoprenaline stimulation (F (1, 11) = 8.357; *P* = 0.0147). Two-way ANOVA with Tukey's post-test. * *p* < 0.05; ** *p* < 0.01; ns: not significant; N.D.: non-detected

Also, we tested whether InRo protocol improves the adipogenic differentiation of SVF monolayers. As shown in Fig. 4A, the mRNA levels of Pparg, Plin1, Adipoq and Lep progressively increased to levels similar to WAT until day 5 of differentiation but showed a decrease thereafter (Fig. 4A). By contrast, AS presented higher mRNA levels of Pparg (2-way ANOVA with Tukey's post-test; *P* = 0.0021), Plin1 (*P* = 0.0055) and Lep (*P* = 0.0012) up to the day 15 of differentiation compared to differentiated adipocyte monolayers.

Next, we compared the abundance of leptin in the conditioned media of differentiated adipocytes monolayers and AS generated in parallel from the same original SVF. Given the variability in sizes and therefore in the number of cells contained in the spheroids and monolayers, leptin concentration was normalized to total DNA content. We found a significant effect of differentiation time (two-way ANOVA; F (3, 24) = 4.375; *P* = 0.0136) and culture format (monolayers vs. AS) (F (1, 24) = 5.948; *P* = 0.0225) on leptin concentration, with no significant interactions between both factors (F (3, 24) = 1.029; *P* = 0.3974). Indeed, in both AS and monolayers leptin concentration increased over time, although the highest concentrations were observed in AS (Fig. 4B). Although no significant time point differences were observed on Tukey post-test, the effect of the culture format was especially noticeable after 15 days of differentiation, when leptin tended to decrease in adipocyte monolayers, in consistency with their lower leptin mRNA levels at this time point, but remained stable in differentiated AS.

To test whether continuous adipogenic stimulation was required for leptin secretion, differentiated monolayers and AS, generated in parallel from the same original SVF, were deprived of insulin and rosiglitazone for 48 h. Following this period, cultures were incubated for additional 48 h with DMEM-F12 and 10% FBS with or without 2,000 nM insulin. Importantly, after adipogenic deprivation leptin was only detected in cultured AS and was even higher than the observed after 15 days of differentiation (0.58 ± 0.36 ng / µg DNA with insulin or 0.57 ± 0.31 ng / µg DNA without insulin compared to 0.30 ± 0.22 ng / µg of DNA, two-way ANOVA; culture format: F (1, 12) = 21,93; *P* = 0,0005; Insulin: F (1, 12) = 1,746e-005; *P* = 0,9967; Interaction: F (1, 12) = 0,01,625; *P* = 0,9007), indicating that AS are able to preserve their adipose phenotype even after a prolonged deprivation of adipogenic stimuli in vitro (Fig. 4C).

A key physiological feature of WAT is its ability to release free fatty acids and glycerol in response to adrenergic stimulation as a result of the lipolytic hydrolysis of intracellular triglycerides, providing substrates for mitochondrial fatty acid beta-oxidation and gluconeogenesis [39, 40]. We assessed the ability of AS differentiated with InRo protocol to emulate this physiological response. For this, we measured glycerol released to the culture medium in response to isoprenaline in AS and monolayers of differentiated adipocytes derived from the same original SVF. As shown in Fig. 4D, we found a significant increase in the extracellular glycerol after isoprenaline stimulation that was ~ twofold higher in differentiated AS in comparison with adipocytes in monolayers, indicating that AS have preserved lipolytic responsivity to beta-adrenergic stimulation.

In conclusion, inRo protocol resulted in the generation of AS able to secrete leptin at higher levels than monolayers of differentiated adipocytes and to respond to a classical lipolytic stimulus to release glycerol to the extracellular medium. Importantly, leptin secretory capacity persisted even after 48 h of adipogenic stimuli deprivation, indicating a stable adipose phenotype and an improved potential to release adipokines in vitro and, eventually, after implantation in vivo.

### Viability and internal ultrastructure of differentiated AS

**Fig. 5:**
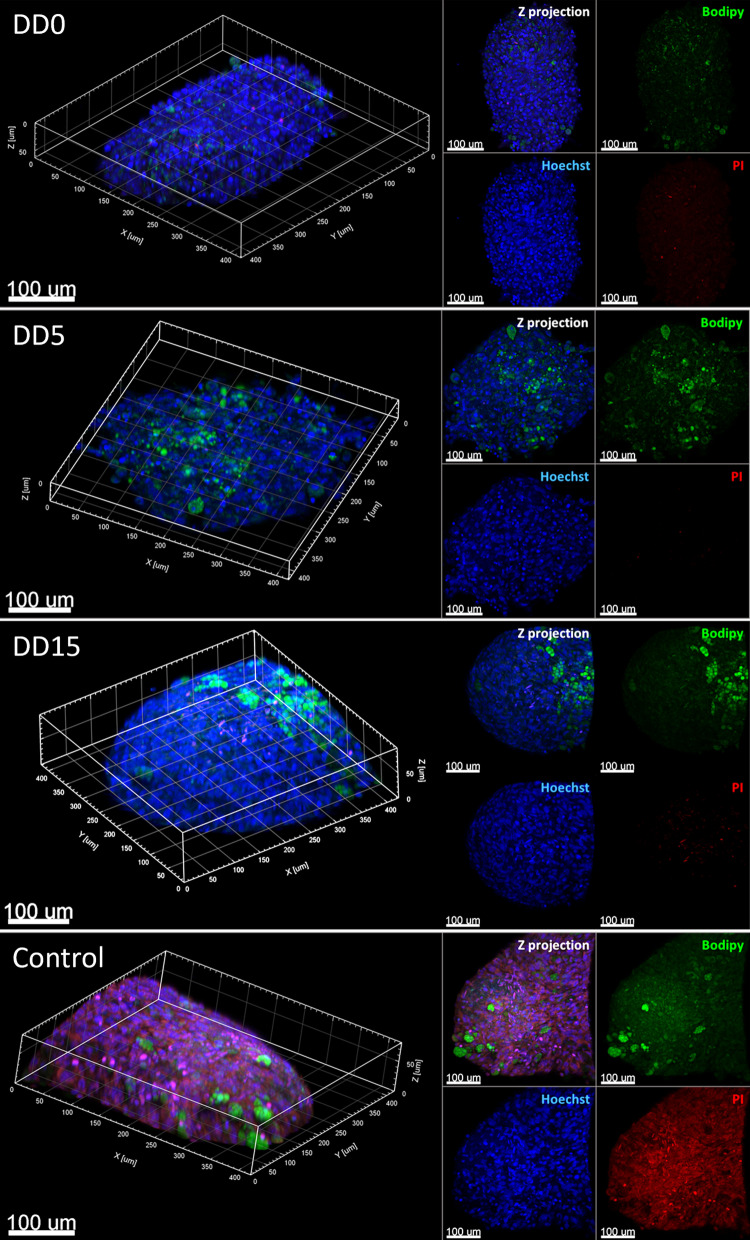
Three-dimensional structure and viability of AS differentiated with InRo protocol. SVF spheroids were subjected to adipogenic differentiation with the InRo protocol for 0, 5, and 15 days (DD0 to DD15, respectively). AS were stained for neutral lipids (Bodipy, green), dead cells (propidium iodide, PI, red) and nuclei (Hoechst, blue). Positive controls for PI staining were post-fixation AS permeabilized with Triton X-100 0.05%. 3D reconstruction and Z-projection based on Z-Stack is shown. Major divisions in 3D reconstruction grid and scale bar of 100 µm

**Fig. 6:**
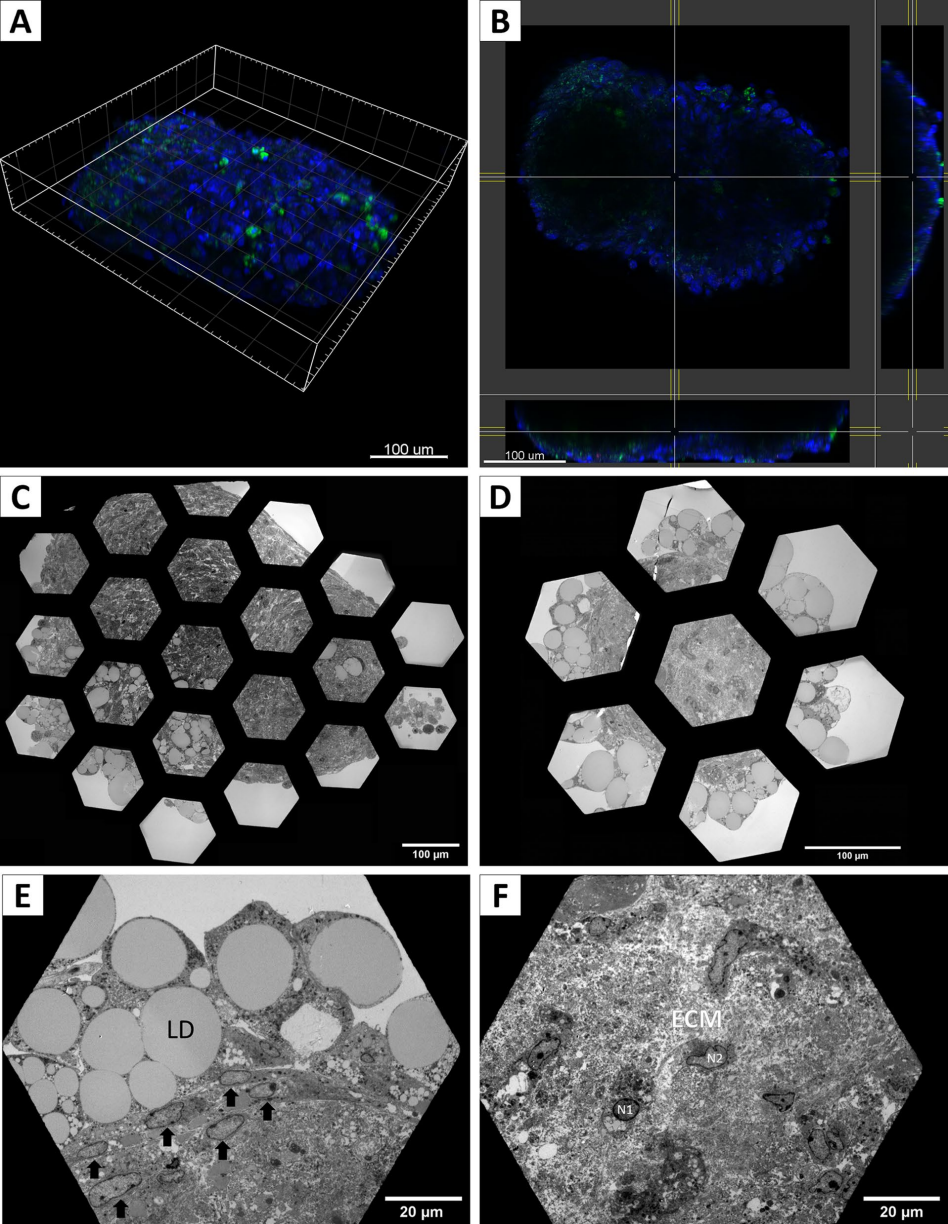
AS differentiated with the InRo protocol have a complex inner structure. **A** 3D reconstruction of confocal Z-stack of AS at day 15 of adipogenic differentiation with InRo protocol and stained for nuclei (Hoechst, blue) and neutral lipid (Bodipy, green) detection. **B**–**F** Ultrastructural analysis of AS at day 15 of differentiation with InRo protocol. (**C** and **D**) Reconstruction of cross-sectioned AS with a montage of images obtained with a magnification of 390x (scale bar of 100 µm). **E** Adipocytes with single and large lipid droplets (LD) in the periphery of the spheroids. Elongated cells (arrow) are organized in 1 to 3 layers of lipid-laden cells. **F** The center of AS shows no evidence of necrosis and cells with small lipid droplets (nucleus N1) or no lipid build up (nucleus N2) are embedded in abundant loose cellular matrix (ECM). Images in (E) and (F) were captured at 790 × magnification, 20 µm scale bar

To assess AS viability, we first determined plasma membrane indemnity by propidium iodide (PI) staining and confocal microscopy analysis. As shown in Fig. 5, only a few positive nuclei were stained with PI, whereas detergent-permeabilized AS showed homogeneous nuclear PI staining indicating proper penetration of this stain into permeabilized spheroids (Fig. 5). Therefore, these results indicate a high level of cellular viability during adipogenic differentiation of AS. Nevertheless, it is important to note that confocal microscopy only allows the evaluation of the outermost layers of AS (Fig. 6A and B), given its maximal penetration depth of ~ 50 µm [41, 42].

To determine the viability of the innermost layers and to characterize the internal ultrastructure of AS, we performed transmission electron microscopy (TEM) analysis on sectioned AS on day 15 of differentiation. As shown in Fig. 6, differentiated spheroids have a complex internal structure, with lipid-laden and lipid-free cells included in abundant extracellular matrix (ECM) (Fig. 6C and D). Higher magnification showed a stratified organization with large unilocular cells present in the outermost layers of the AS (Fig. 6E, "LD"). Immediately under this adipocyte-like layer, there were 2 to 3 layers of elongated cells devoid of lipid droplets, resembling undifferentiated mesenchymal cells or fibroblasts [9, 14] (Fig. 6E, arrow). Towards the center of spheroids, lipid-laden cells were smaller compared to adipocyte-like cells present in the AS periphery (Fig. 6F, “N1”) and were intermixed with lipid droplets-free cells and loose ECM (Fig. 6F, “ECM”).

**Fig. 7:**
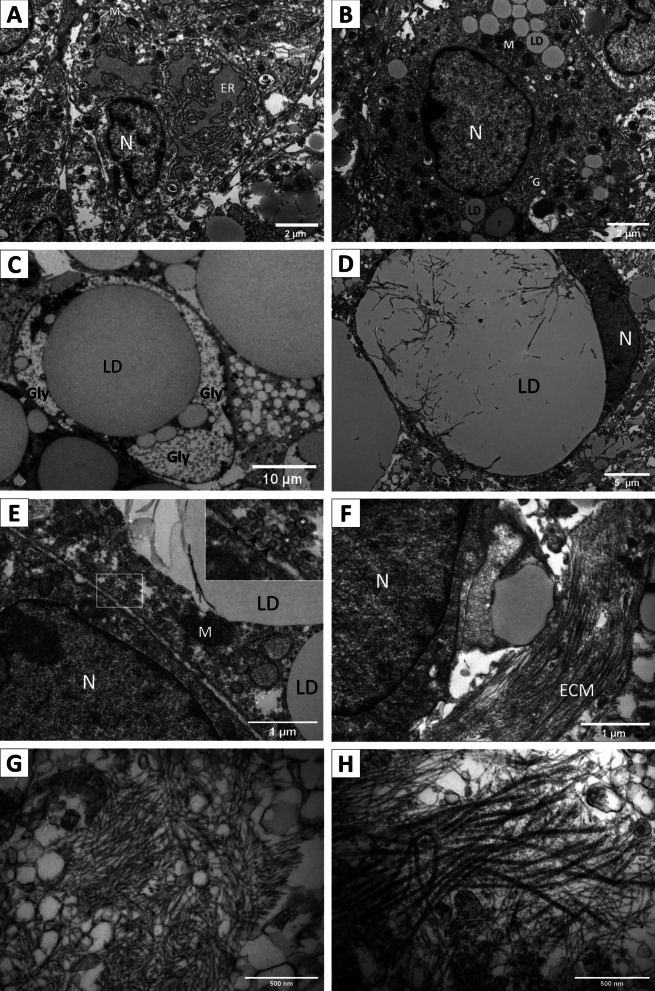
Ultrastructural analysis of differentiated AS reveals a multilayer organization with different cell types and an organized extracellular matrix. **A** Elongated cells with few rounded mitochondria, extensive endoplasmic reticulum with distended cisternae and cytoplasmic vesicles (asterisk) likely correspond to undifferentiated preadipocytes. **B** Small lipid droplets towards the cellular poles of putative preadipocytes. 2 µm bar in A and B. **C** Adipocytes in advanced differentiation stages are characterized by glycogen granules near the lipid droplets, rounded cell shape, and displacement of the nucleus towards the cellular periphery. 10 µm scale bar. **D** Terminally differentiated adipocytes are characterized by a single giant lipid droplet and a “ring-shaped” cytoplasm surrounding it, with a crescent moon-shaped nucleus on the one side of the lipid droplet. 5 µm scale bar. **E** The contact zone between plasma membranes of two adjacent adipocytes shows abundant and well-defined individual caveolae and caveolae/clusters known as rosettes. Frame magnification in E showing 3 individual caveolae (black arrow) and two rosettes (white asterisk). **F** The extracellular matrix in the adjacent zone to the adipocyte shown in panel (D) shows thick and banded fibers with a parallel arrangement, interspersed by thinner fibers with a looser disposition. 1 µm scale bar in E and F. **G**-–**H** Towards the center of AS is abundant ECM with a loose appearance, with thin and dispersed fibers (G) intertwined with thicker and banded fibers (H). 500 nm scale bar in G and H. N, nucleus; ER, endoplasmic reticulum; M, Mitochondrion; LD, lipid droplet; G, Golgi complex; Gly, glycogen; ECM, extracellular matrix

Abundant cells with large, pale, and irregular nuclei and prominent nucleoli were present (Fig. 7A). In these cells, the endoplasmic reticulum was prominent (Fig. 7A, "ER") and mitochondria were small and spherical (Fig. 7A, “M”). Also, these cells had abundant vesicles at the cytoplasmic periphery (Fig. 7A, asterisk). These features are reminiscent of the described ultrastructure of human MSC in muscle [43], bone marrow [44], subepicardial adipose tissue [45], and rat MSC [46].

Other cells showed numerous small lipid droplets (Fig. 7B, "LD") and scarce endoplasmic reticulum. Mitochondria were mostly spherical but occasionally elongated (Fig. 7B, "M"), the Golgi apparatus was well-defined (Fig. 7B, "G") and cytoplasmic vesicles were abundant. Glycogen granules were also present, mainly in the vicinity of large lipid droplets (Fig. 7C, “Gly”). As these ultrastructural features have been described in differentiating rat adipocytes [47], we propose that these cells correspond to adipocytes at the early phases of differentiation.

It is important to note that differentiated adipocytes in the outer layers of AS were unilocular, i.e., harbor a single giant lipid droplet, with the nucleus and cytoplasm displaced to the margin of the plasma membrane (Fig. 7D). In these cells, plasma membrane caveolae were abundant, especially in areas of intercellular contact (Fig. 7E). Unilocular morphology and abundant caveolae are both key morphological features of mature adipocytes that are rarely seen in classical bidimensional cultures of differentiated adipocytes.

ECM was particularly abundant at the center of AS, and it was formed by parallel fibers in close contact with both lipid-laden and lipid-free cells. Frequently, a combination of at least two types of ECM fibers was noted (Fig. 7F). In low cellularity areas, especially at the center of AS, ECM was composed either of fine and dispersed fibers (Fig. 7G) or thicker and banded fibers with a parallel organization (Fig. 7H). This is consistent with elastic fibers intermixed with collagen fibers; however, the molecular identity of ECM in AS remains to be determined.

## Discussion

Herein we show that the SVF harvested from the interscapular adipose tissue of newborn mice is composed of mesenchymal cells with a differentiation potential toward adipocyte and chondrocyte but not osteoblast linage. We also show that this SVF can be used for AS formation either by the hanging drops or the low adherence plate method, and the latter yields the higher number and the biggest spheroids. Finally, maintaining rosiglitazone throughout the entire adipogenesis protocol results in highly viable, leptin-secreting, and lipolysis-responsive AS that also have a complex internal structure. In fact, AS consists of adipocyte precursors, adipocytes at various stages of differentiation, terminally differentiated unilocular adipocytes, as well as non-adipose cell types, all of them embedded in an organized extracellular matrix. At the molecular level, AS express mature adipocyte markers at levels equivalent to the WAT of adult mice, secrete leptin even after deprivation of adipogenic pharmacological stimulus, and release glycerol in response to stimulation with isoprenaline, indicating a preserved capacity to hydrolyze triglycerides in response to beta-adrenergic activation.

Available methods to form multicellular spheroids include: (1) cellular pellets, (2) culture under continuous shaking or rotation, (3) hanging drop culture, (4) low-adherence surface culture, (5) culture in the presence of external forces (electromagnetic field or ultrasound) and (6) microfluidic chambers or micro-molds [12]. Herein, we assessed hanging drop and low-adherence culture methods because they involve lower mechanical stress that otherwise may destroy adipocytes, given their intrinsic mechanical fragility and high buoyancy. We found that the formation of spheroids in low-adherence plates yields abundant (~ 2,00 spheroids per newborn mice) and large-sized (~ 300 μm) spheroids, suitable for in vitro and in vivo experimentation in mice.

During adipogenic differentiation, numerous small lipid droplets fuse to form a single large lipid droplet that displaces the nucleus and other organelles towards the cell periphery [47]. Although this morphological feature is a defining feature of mature white adipocytes, classical adipogenic protocols based on monolayer cultures fail to form unilocular cells. This outcome is likely the result of incomplete adipogenesis because of the limited time that lipid-laden cells can be maintained in monolayers. In fact, after day 7 of differentiation, we found a systematic and highly reproducible decrease in the abundance of mature adipocyte markers, as well as, in the proportion of lipid-laden cells. It is plausible that this simply occurs by the selective detachment of highly buoyant lipid-laden cells in this culture format. By contrast, given the cohesive structure of AS, this culture format offers multiple cell-to-cell and cell-to-ECM attaching sites, allowing the retention of fully differentiated-unilocular adipocytes.

Adipose tissue secretes more than 600 peptide hormones (adipokines) with diverse endocrine, autocrine, and paracrine actions. Leptin was the first adipokine to be described and has a central role in energy, lipid, and carbohydrate metabolism [48–51]. Nevertheless, the only approved therapeutical application for leptin is the control of metabolic complications in patients with genetic leptin deficiency or in patients with severe adipose tissue restriction (lipodystrophy) [52–55]. In these patients, leptin supplementation ameliorates insulin resistance, hyperglycemia, hypertriglyceridemia, hepatic steatosis, and female infertility [53, 56–58]. However, leptin supplementation remains unavailable in most low- and middle-income countries, and it requires lifelong daily injections, making it unaffordable in most cases. It is possible that implantation of heterologous or even autologous AS (after gene editing of pathogenic mutations) could offer a therapeutic alternative for leptin-deficient patients.

## Conclusions

In this work, we developed a method for the efficient production of AS from the SVF of the mouse interscapular adipose tissue. These structures are composed of fully differentiated white adipocytes and a variety of other cells organized in a complex adipose organoid that can sustain prolonged adipogenic differentiation protocols in vitro and secrete leptin and undergo lipolysis after stimulation with insulin and beta-adrenergic agonists, respectively. Importantly, AS can resume leptin secretion after deprivation of adipogenic stimulus, indicating that they are suitable for implantation in animals and potentially sustain leptin production in mouse models of generalized lipodystrophy or genetic leptin deficiency.

## Supplementary Information

**Additional file 1.** Complete set of Image J scripts used for the geometrical analysis of adipose spheroids.

**Additional file 2.** Nucleotide sequence of PCR primers used in this work.

**Additional file 3.** **Supplemental Figure 1** Characterization of adipocyte monolayers and AS differentiated with classical “i20” protocol. (A) Representative confocal image of cross-sectioned AS (30 µm thickness) at day 10 of adipogenic differentiation, stained for neutral lipids (Bodipy, green) and nuclei (Hoechst, blue). Scale bar 100 µm. (B) Gene expression levels of adipocyte markers in differentiated adipocyte monolayers (blue) and AS (green). mRNA abundance is expressed as fold-change to the abundance of the corresponding mRNAs in adult mouse WAT (dotted line). Values correspond to mean and standard deviation, N = 4 per group. Two-way ANOVA with Tukey's post-test. *p<0.05; ** p < 0.01; ns not significant.

**Additional file 4.** **Supplemental Figure 2** Effect of insulin on the adipogenic differentiation of SVF derived spheroids.(A) SVF spheroids cultures were differentiated for 15 days with an adipogenic cocktails with growing insulin concentrations (i0 to i10,000 correspond to 0, 20, 2,000 or 10,000 nM of insulin, respectively). Pparg, Plin1, Adipoq and Lep mRNA levels were expressed as fold-change relative to adipocytes differentiated with the classic cocktail (i20, dotted line). Each bar corresponds to a pool of 4 independent cultures. (B) Leptin concentration in conditioned medium (final 48 hours) at day 15 of differentiation, with 100 spheroids per group, N = 3. One-way ANOVA (F (3, 8) = 7.307, p = 0.0111). * p < 0.05; ns: not significant; N.D. not detected.

## Data Availability

All the row data, including images and quantitative data, are available under request to the corresponding author (Victor Cortes, vcortesm@uc.cl).

## Abbreviations

Adipoq: Adiponectin
ASCs: Adipose tissue-derived stromal cells
AS: Adipose spheroids
ANOVA: Analysis of variance
2D: Bidimensional monolayer
Cebpb: CCAAT/enhancer-binding protein beta
ECM: Extracellular matrix
FBS: Fetal bovine serum
Lep: Leptin
MSC: Mesenchymal stem cells
Plin1: Perilipin 1
Pparg: Peroxisome proliferator-activated receptor gamma
3D: Three-dimensional
TEM: Transmission electron microscopy
WAT: White adipose tissue
IBMX: 3-Isobutyl-1-methylxanthine
