# Supplementary material for: Spheroids derived from the stromal vascular fraction of adipose tissue self-organize in complex adipose organoids and secrete leptin
Source: Stem Cell Res Ther. 2023 Apr 7;14:70. doi: 10.1186/s13287-023-03262-2 (PMC10080976; doi:10.1186/s13287-023-03262-2)
Supplement: Supplementary file 1 — Additional file 1. Complete set of Image J scripts used for the geometrical analysis of adipose spheroids. [file 13287_2023_3262_MOESM1_ESM.docx]

Supplemental Table 1. Image J Script

//setTool("line");

makeLine(272, 812, 2364, 828);

run("Set Scale...", "known=1000 unit=um global");

close();

run("Labels...", "color=white font=24 show");

name=getTitle;

run("Duplicate...", "title=Blur");

run("Gaussian Blur...", "sigma=300");

imageCalculator("Divide create 32-bit", name,"Blur");

run("8-bit");

setAutoThreshold("Default");

//run("Threshold...");

//setThreshold(0, 91);

run("Convert to Mask");

run("Fill Holes");

run("Analyze Particles...", "size=2000-Infinity circularity=0.10-1.00 show=Overlay display exclude");

run("Set Measurements...", "area shape feret's display add redirect=None decimal=2");
