# Supplementary material for: Spheroids derived from the stromal vascular fraction of adipose tissue self-organize in complex adipose organoids and secrete leptin
Source: Stem Cell Res Ther. 2023 Apr 7;14:70. doi: 10.1186/s13287-023-03262-2 (PMC10080976; doi:10.1186/s13287-023-03262-2)
Supplement: Supplementary file 2 — Additional file 2. Nucleotide sequence of PCR primers used in this work. [file 13287_2023_3262_MOESM2_ESM.docx]

Supplemental Table 2. RT-qPCR primer set

| Gene name | NCBI reference | Sense primer | Antisense primer |
| --- | --- | --- | --- |
| Cebpb | NM_001287738.1 | cccgtacgccaggcagt | cgcctttagacccatggaag |
| Pparg | NM_001308354.1 | cacaatgccatcaggtttgg | gctggtcgatatcactggagatc |
| Plin1 | NM_001113471.1 | gtggagcgggacctgtga | ttctcataggcattgcacacaga |
| Adipoq | NM_009605.5 | tcacggtgtacatgaaagatgtg | gagaacggccttgtccttct |
| Lep | NM_008493.3 | ctccatctgctggccttctc | catccaggctctctggcttct |
| cyclophilin | NM_011149.2 | tggagagcaccaagacagaca | tgccggagtcgacaatgat |
