# Supplementary figures and images for: Spheroids derived from the stromal vascular fraction of adipose tissue self-organize in complex adipose organoids and secrete leptin
Source: Stem Cell Res Ther. 2023 Apr 7;14:70. doi: 10.1186/s13287-023-03262-2 (PMC10080976; doi:10.1186/s13287-023-03262-2)

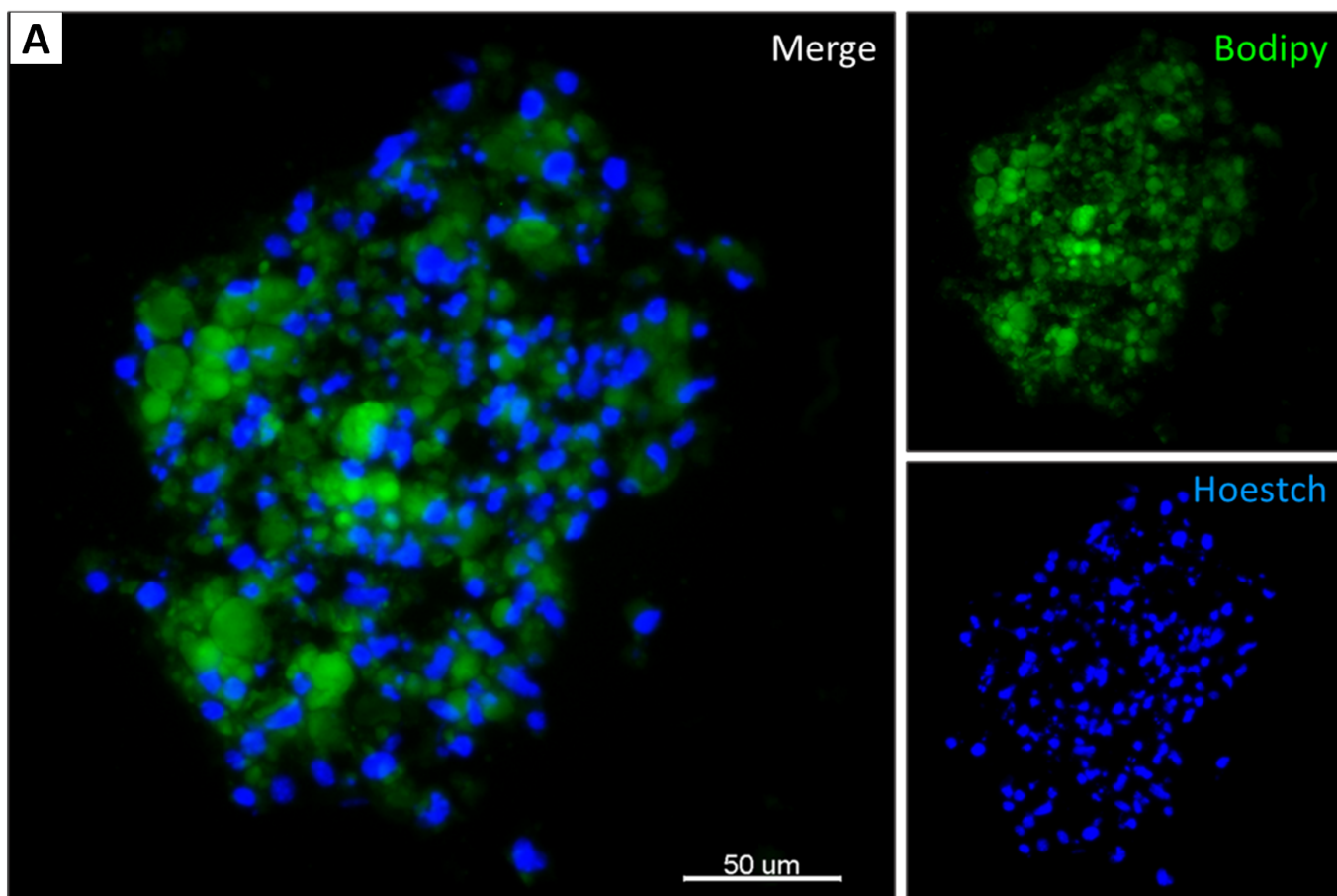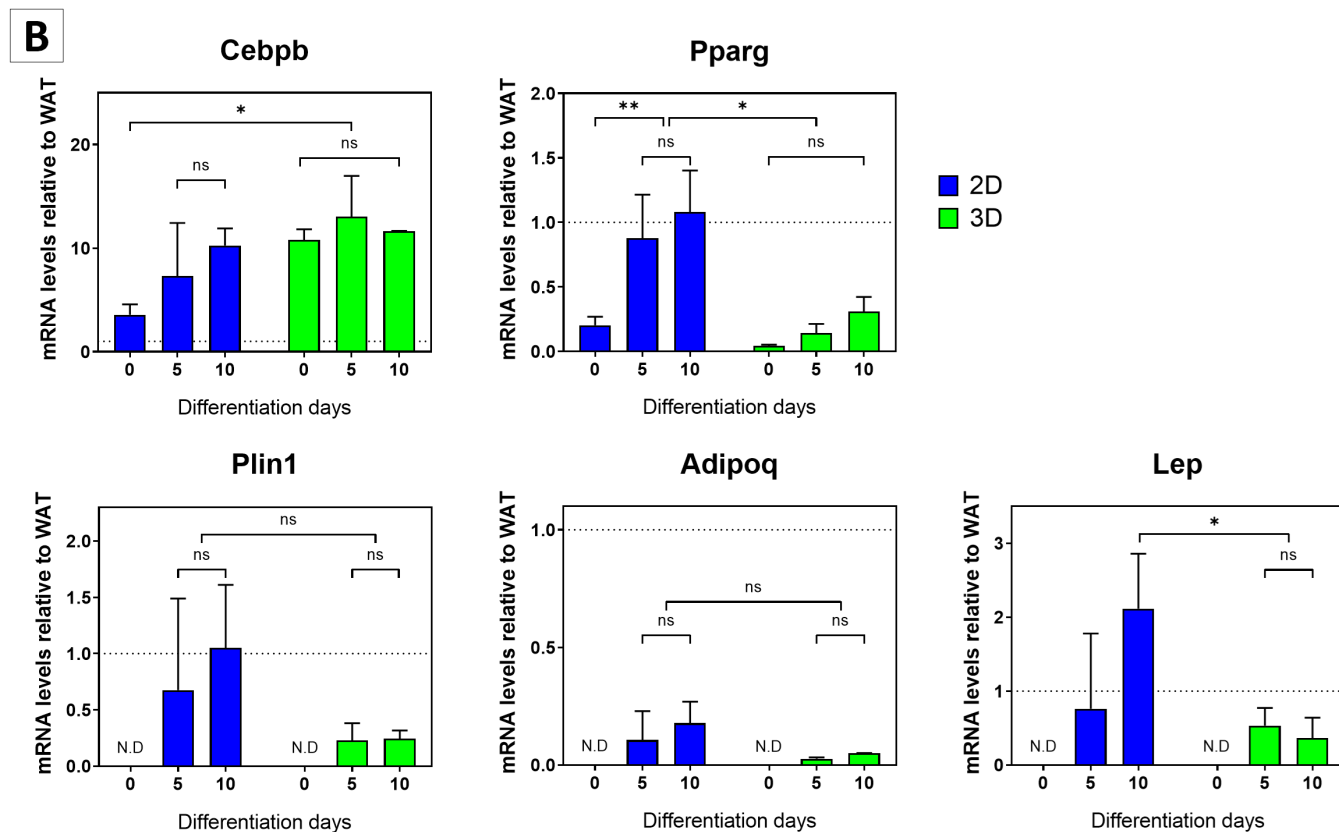

Supplement: Supplementary file 3 — Additional file 3. Supplemental Figure 1 Characterization of adipocyte monolayers and AS differentiated with classical “i20” protocol. (A) Representative confocal image of cross-sectioned AS (30 µm thickness) at day 10 of adipogenic differentiation, stained for neutral lipids (Bodipy, green) and nuclei (Hoechst, blue). Scale bar 100 µm. (B) Gene expression levels of adipocyte markers in differentiated adipocyte monolayers (blue) and AS (green). mRNA abundance is expressed as fold-change to the abundance of the corresponding mRNAs in adult mouse WAT (dotted line). Values correspond to mean and standard deviation, N = 4 per group. Two-way ANOVA with Tukey's post-test. *p<0.05; ** p < 0.01; ns not significant. [file 13287_2023_3262_MOESM3_ESM.pdf]
