# Supplementary material for: Spheroids derived from the stromal vascular fraction of adipose tissue self-organize in complex adipose organoids and secrete leptin
Source: Stem Cell Res Ther. 2023 Apr 7;14:70. doi: 10.1186/s13287-023-03262-2 (PMC10080976; doi:10.1186/s13287-023-03262-2)
Supplement: Supplementary file 4 — Additional file 4. Supplemental Figure 2 Effect of insulin on the adipogenic differentiation of SVF derived spheroids.(A) SVF spheroids cultures were differentiated for 15 days with an adipogenic cocktails with growing insulin concentrations (i0 to i10,000 correspond to 0, 20, 2,000 or 10,000 nM of insulin, respectively). Pparg, Plin1, Adipoq and Lep mRNA levels were expressed as fold-change relative to adipocytes differentiated with the classic cocktail (i20, dotted line). Each bar corresponds to a pool of 4 independent cultures. (B) Leptin concentration in conditioned medium (final 48 hours) at day 15 of differentiation, with 100 spheroids per group, N = 3. One-way ANOVA (F (3, 8) = 7.307, p = 0.0111). * p < 0.05; ns: not significant; N.D. not detected. [file 13287_2023_3262_MOESM4_ESM.pdf]

A

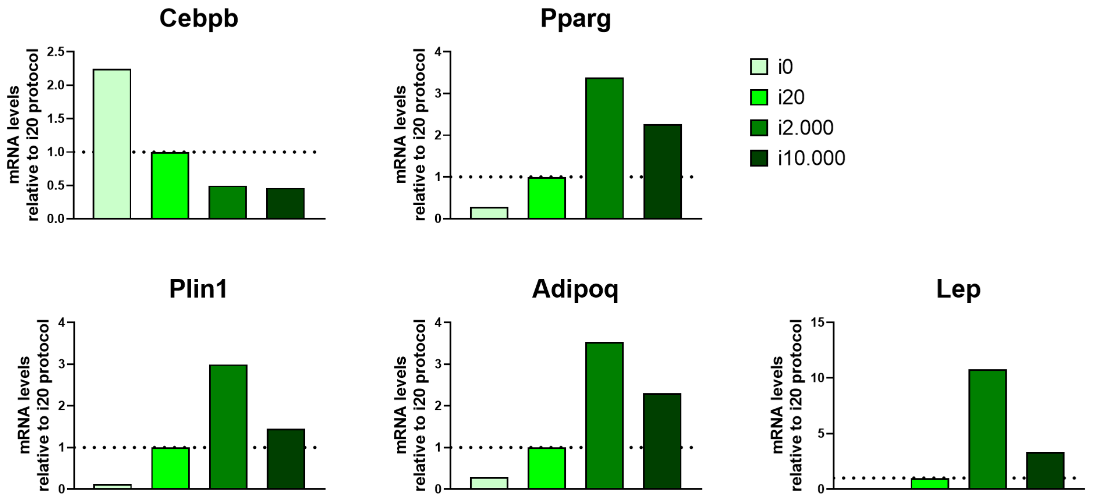

B

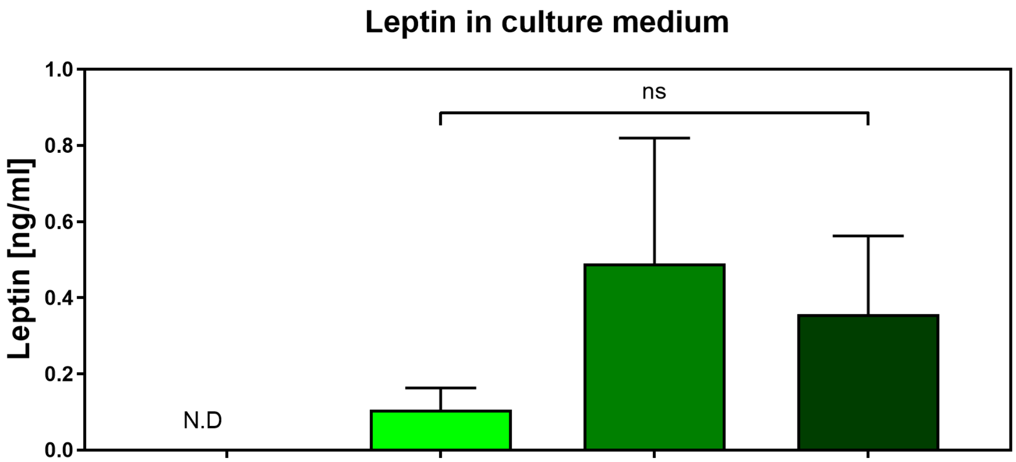

Medium	i0	I20	I2,000	I10,000
Dexamethasone [nM]	500	500	500	500
Indomethacin [nM]	125	125	125	125
IBMX [mM]	0.5	0.5	0.5	0.5
Rosiglitazone [nM]	1.0	1.0	1.0	1.0
T3 [nM]	1.0	1.0	1.0	1.0
Insulin [nM]	0	20	2,000	10,000
